# Editorial Note: Expression of Concern: Chaperone-mediated autophagy targets IFNAR1 for lysosomal degradation in free fatty acid treated HCV cell culture

**DOI:** 10.1371/journal.pone.0338988

**Published:** 2025-12-17

**Authors:** The PLOS One Editors

After publication of this article [1] and the related Expression of Concern [2], concerns were raised with the methodology used in [1]. Specifically, that the siRNA sequence to knock down LAMP2A in human-derived Huh-7.5 cells contains mismatches, including one in the seed region, such that it is not a match for the human mRNA sequence, and is instead a near-match for the mouse mRNA.

The corresponding author stated that the siRNA for Lamp2A was purchased from Invitrogen and is predesigned and validated for Lamp2A. They stated that they did not check whether the sequence was a human or a mouse but noted that keeping two UU at the 3’ end improves the siRNA performance and that this siRNA is used to silence LamP2A in human cells [3,4]. The corresponding author stated that the Lamp2A siRNA used in [1] has six nucleotides that are the same as the human seed sequence and one mismatch in the seventh nucleotide. They also stated that Lamp2A siRNA matched to the mouse sequence is effective for silencing LAMP2A in human cells and avoiding off-target issues.

Members of the *PLOS One* Editorial Board reviewed the author’s response to the above methodology concern and stated that the explanation for the 3’ mismatch is satisfactory and that LAMP2 silencing has also been assessed by western blotting in Fig 10D in [1]. However, they also noted that while such mismatches in the seed region may sometimes allow functional silencing, this needs to be balanced with standard expectations in validating gene knockdown studies.

Members of the *PLOS One* Editorial Board raised an additional concern with the methodology in [1], that no rescue experiment (an siRNA-resistant LAMP2A construct would be introduced to reverse the observed phenotype) is performed in [1] which is needed to exclude off-targets effect due to siRNA transection and therefore this is a major limitation in establishing causality. They noted that [1] includes a scrambled siRNA control, but this does not rule out sequence-specific off-target silencing.

A member of the *PLOS One* Editorial Board stated that the absence of a rescue experiment in [1] raises concerns about the strength of the mechanistic claims, however, the findings may still be valid and hypothesis-generating if future confirmatory experiments are undertaken.

This notice updates the reasons underlying the Expression of Concern [2].

In addition, after publication of this article [1] and the related Expression of Concern [2], concerns were raised with Figs 5 and 7. Specifically:

- The HCV + FFA ß-actin of Fig 5A of [1] appears similar to the Z-IETD-FMK TOV-21G - ß-actin panel in Fig 3A in a later article [5], retracted in [6].
- The ß-actin panel of Fig 7A of [1] appears similar to the Z-IETD-FMK ES-2 + ß-actin panel in Fig 3A in a later article [5], retracted in [6].

The concerns with Figs 5 and 7 are not included in the reasons underlying the Expression of Concern [2].

## References

[1] Kurt R, Chandra PK, Aboulnasr F, Panigrahi R, Ferraris P, Aydin Y, et al. Chaperone-Mediated Autophagy Targets IFNAR1 for Lysosomal Degradation in Free Fatty Acid Treated HCV Cell Culture. PLoS One. 2015;10(5):e0125962. doi: 10.1371/journal.pone.0125962 25961570 PMC4427131

[2] The *PLOS One* Editors. Expression of Concern: Chaperone-mediated autophagy targets IFNAR1 for lysosomal degradation in free fatty acid treated HCV cell culture. PLoS One. 2025;20(2):e0319262. doi: 10.1371/journal.pone.0319262 39919083 PMC11805412

[3] Matsui C, Deng L, Minami N, Abe T, Koike K, Shoji I. Hepatitis C Virus NS5A Protein Promotes the Lysosomal Degradation of Hepatocyte Nuclear Factor 1α via Chaperone-Mediated Autophagy. J Virol. 2018;92(13):e00639-18. doi: 10.1128/JVI.00639-18 29695419 PMC6002715

[4] Saha T. LAMP2A overexpression in breast tumors promotes cancer cell survival via chaperone-mediated autophagy. Autophagy. 2012;8(11):1643–56. doi: 10.4161/auto.21654 22874552 PMC3494593

[5] McGlorthan L, Paucarmayta A, Casablanca Y, Maxwell GL, Syed V. Progesterone induces apoptosis by activation of caspase-8 and calcitriol via activation of caspase-9 pathways in ovarian and endometrial cancer cells in vitro. Apoptosis. 2021;26(3–4):184–94. doi: 10.1007/s10495-021-01657-1 33515314

[6] McGlorthan L, Paucarmayta A, Casablanca Y, Maxwell GL, Syed V. Retraction Note: Progesterone induces apoptosis by activation of caspase-8 and calcitriol via activation of caspase-9 pathways in ovarian and endometrial cancer cells in vitro. Apoptosis. 2025;30(1–2):506. doi: 10.1007/s10495-024-02062-0 39738841

